# Spanish Review

**Published:** 1891-05

**Authors:** J. O’Leary


### *SPANISH.*

BY J. O'LEARY M. D.

---

### SOME DISEASES OF LAMBS.

*Constipation.*—One of the first ailments to which lambs are liable soon after birth is constipation, not however as this condition is usually understood. The first evacuations are of an adhesive character and stick to the wool in the neighborhood of the anus, so that thereafter the poor little animal ceases to have any movement from the bowels. When a lamb is noticed to be out of sorts, and has been carefully examined, this condition of things is often found to exist. The remedy consists in washing the parts and clipping the wool around the auus. It is often found however that lambs suffer from real constipation. This may be due to the deprivation of the mother's milk, which after parturition contains purgative elements. Relaxing food should be given them instead of dry forage. If this should not succeed a little Glauber's salts may be tried.

*Diarrhœa.*—This disease is much more frequent among lambs than the one above mentioned. Very often the mother's milk is too strongly purgative, and for some time after birth they will suffer from profuse discharges from the bowels. This may also be due to improper food. The treatment consists in regulating the diet, taking the lamb from the mother if necessary. For some time nothing but warm milk with lime water should be allowed.

*Lameness.*—Lambs are liable to a peculiar and frequently fatal disease, the chief feature of which is lameness. This disease generally attacks them within the first eight months after birth. Its results are fatal to one half of the flock. Those attacked seem at first sad and, after lying down, are unable to rise again. The limbs become rigid, sometimes the front ones only; sometimes the hind ones only; at other times the rigidity extends over the entire body. Swellings make their appearance, especially in the joints. Sometimes there is an eruption in the skin of the nature of mange. Diarrhœa soon sets in quickly followed by death. The cause of the disease is not known, nor have the means of prevention or cure been ascertained. *Gaceta Medico-Veterinaria, Madrid.*

